# Family-Based WhatsApp Intervention to Promote Healthy Eating Behaviors Among Amazonian School Children: Protocol for a Randomized Controlled Trial

**DOI:** 10.2196/54446

**Published:** 2024-02-19

**Authors:** Ana Carolina de Andrade Hovadick, Marly Augusto Cardoso

**Affiliations:** 1 Department of Nutrition, School of Public Health University of Sao Paulo Sao Paulo Brazil; 2 Global Health and Tropical Medicine (GHTM), Associate Laboratory in Translation and Innovation Towards Global Health (LA-REAL), Instituto de Higiene e Medicina Tropical (IHMT) Universidade NOVA de Lisboa (UNL) Lisbon Portugal

**Keywords:** child health, Amazon, dietary intake, mHealth, mobile health, multimedia messaging service, WhatsApp

## Abstract

**Background:**

Stunting and micronutrient deficiencies have persistently affected children in the Brazilian Amazon for decades. However, in recent years, a notable increase in childhood overweight prevalence has been observed, particularly in the context of heightened food insecurity exacerbated by the COVID-19 pandemic. Despite the limited number of effective solutions proposed to tackle this problem, digital interventions have shown great promise worldwide in preventing obesity and promoting healthy diets.

**Objective:**

This study aims to describe the protocol of a family-based WhatsApp intervention, specifically designed to investigate the efficacy of multimedia messaging in preventing excessive weight gain and improving healthy eating practices among school-aged children in the Amazon region.

**Methods:**

This study protocol outlines a theory-driven randomized controlled trial based on the cognitive theory of multimedia learning and the social cognitive theory. A total of 240 parents or caregivers of children enrolled in the Maternal and Child Health and Nutrition Cohort Study in Acre (MINA-Brazil) will be recruited by phone and social media. The intervention group will receive persuasive multimedia messages through WhatsApp over 19 weeks, while the waitlist control group will remain in the usual care. The primary outcome is a change in children’s BMI in *z* score. Secondary outcomes are changes in dietary intake and biochemical indicators of the children. Outcome measures will be assessed at baseline and 5 months after randomization in comparison to usual care. The analysis will use an intent-to-treat approach and will be conducted using the statistical package Stata (version 18.0), with a significance level set at *P*<.05. Paired and unpaired 2-tailed *t* tests will be applied to compare mean changes in the outcomes.

**Results:**

Data collection started in June 2023, and final measurements are scheduled to be completed in December 2023. The results of the main analysis are expected to be available in 2024.

**Conclusions:**

This innovative multimedia message intervention holds significant potential for fostering behavioral changes among Amazonian children.

**Trial Registration:**

Brazilian Clinical Trials Registry RBR-5zdnw6t; https://ensaiosclinicos.gov.br/rg/RBR-5zdnw6t

**International Registered Report Identifier (IRRID):**

DERR1-10.2196/54446

## Introduction

In recent decades, childhood obesity has emerged as a global epidemic [1]. According to the World Health Organization (WHO), more than 340 million children were overweight or obese in 2016 [2]. In addition, with the emergence of the COVID-19 pandemic in 2019, the situation has been aggravated due to school closures, physical inactivity, dietary changes, and mental health disorders [3].

In Brazil, according to the latest national school anthropometric survey, in 2009, 1 out of 3 children were overweight [4]. However, it is expected that due to the COVID-19 crisis and the absence of national child anthropometric surveys for 15 years, the current prevalence of childhood overweight has likely worsened [5,6]. In this context, the Brazilian Amazon was one of the most affected regions in the country due to its remote location and lack of specialized health assistance [5,7]. Recent studies revealed that approximately 70% of the northern population experience food insecurity [8]. Additionally, 20% experience severe food insecurity, wherein individuals feel hungry but do not have funds to buy food, have only 1 meal a day, or endure entire days without any meals [8].

In this scenario, considering the Brazilian economic crisis paired with the neglected fast growth of the ultraprocessed food (UPF) industry, access to fresh food has become challenging, while UPF has become ubiquitous in most food markets [9,10]. UPF are formulations produced exclusively by the food industry through processing techniques that damage the food matrix (eg, extrusion, refining, and prefrying). Additionally, UPFs are frequently high in calories, harmful fats, sugar, and artificial additives [11]. Many studies in the literature have shown that the consumption of UPF may increase the risk of morbidity and early mortality [12]. Therefore, UPF’s high availability and consumption are significantly endorsing a substantial change in Western Brazilian Amazon food patterns, which has placed overweight and obesity as an important public health problem due to the historical rates of stunting and micronutrient deficiencies in the region [13-15]. Although some studies have called attention to the urgent need for early interventions to prevent childhood obesity in the Amazon region, few effective solutions have been proposed to tackle this problem so far [16,17].

Along these lines, digital interventions focused on promoting weight loss and healthy eating behaviors have shown promising results, especially mobile phone interventions (mobile health [mHealth]) [18,19]. MHealth interventions are innovative and have countless advantages. First, it increases the contact between health care professionals and people living in remote areas, such as the Amazon region [20]. Second, it is a low-cost, easy-to-use, and alternative tool that is already widely incorporated into society. Lastly, these interventions can be developed on a large scale, benefiting people in a wide region. Although Amazon is considered a location of poverty and of great inaccessibility, about 80% of the population has access to the internet, and 8 out of 10 adults have a cell phone [21,22].

In this context, multimedia messages have spread rapidly in society in recent years. Since it includes both textual and visual resources, it provides efficient learning due to its greater ability to establish itself in the brain’s long-term memory; therefore, it demonstrates great potential to generate changes in health behaviors, especially in populations with low educational levels [23-26]. As far as we know, no studies have evaluated the effects of multimedia messages on children’s health behaviors; however, there is plenty of evidence indicating the extensive potential of multimedia resources. Randomized controlled trials based on soap operas, video games, and websites as multimedia components have reported a significant increase in fruit and vegetable consumption and a decrease in sugary drinks and sweets consumption [27-33].

Also, a recent systematic review and meta-analysis showed that childhood obesity risk is significantly influenced by parental weight status [34]. Thus, parent-focused interventions are an important key to promoting efficient and lasting changes in children’s eating behaviors. Family-based interventions focusing on children who are overweight that used SMS text messaging lead to a significant reduction in children’s adiposity and BMI [35]. Additionally, healthy eating patterns were improved among all family members [36]. These findings support the hypothesis that family-based multimedia messaging interventions have great potential to promote behavioral changes in children.

This study protocol will be the first to assess the effect of mHealth on dietary practices and anthropometric measurements of Amazonian children. We aim to develop a parenting-focused, mobile-based intervention through multimedia messaging to promote healthy eating habits and prevent obesity among Amazonian children. We also intend to support and provide high-quality evidence to guide future public policy regarding childhood obesity, food insecurity, and sustainable food systems.

## Methods

### Study Aim

The general aim of this study is to evaluate the efficacy of a parent-focused mHealth intervention to change dietary practices and prevent excessive weight gain among school-aged children from the Maternal and Child Health and Nutrition Cohort Study in Acre (MINA-Brazil). Therefore, we hypothesize that children whose parents or caregivers receive nutrition-related multimedia messages will have improvements in BMI in *z* score (BMIz), dietary food intake, and biochemical data.

### Overview and Trial Design

This trial will be a parallel randomized controlled trial nested in MINA-Brazil, the first population-based birth cohort based in Cruzeiro do Sul, Acre State, in the Western Brazilian Amazon. The cohort was designed to investigate the determinants of maternal and child health and have as participants children born at the Hospital Estadual da Mulher e da Criança do Jurua, in Cruzeiro do Sul, in the period between July 2015 and June 2016. Participants have been followed up for 5 years, and the last evaluation of health status took place in 2021. This protocol was prospectively registered in the Brazilian Clinical Trials Registry (RBR-5zdnw6t; date of registration: March 30, 2023; UTN code: U1111-1289-0560) and has been described according to the CONSORT-EHEALTH checklist (version 1.6.1; Multimedia Appendix 1 [37-47]) [48]. Table 1 shows the timeline of the study activities according to the intervention stage:

**Table 1:** Timeline of the study activities according to intervention stages.

Stage of intervention	Recruitment	Baseline	Intervention period	Postintervention
Verbal consent for screening	✓			
Eligibility screening verification	✓			
Study invitation	✓			
Face-to-face individual interview		✓		
Written consent from parents and written assent from children		✓		
Sociodemographic and participant information collection		✓		
Anthropometric measures		✓		✓
Food frequency questionnaire administration		✓		✓
Biochemical data collection		✓		✓
Strengths and Difficulties Questionnaire administration		✓		✓
Short version of the Brazilian Food Insecurity Scale administration		✓		✓
Delivery of an educational booklet		✓		
Goals setting for intervention group			✓	
Multimedia messaging for a 19-week follow-up			✓	
Phone call at 1 week to check if caregivers are properly receiving the messages			✓	
Goal achievement assessment for intervention group				✓
Delivery of same intervention to waitlist control group.				✓

### Study Population and Setting

**Figure 1:**
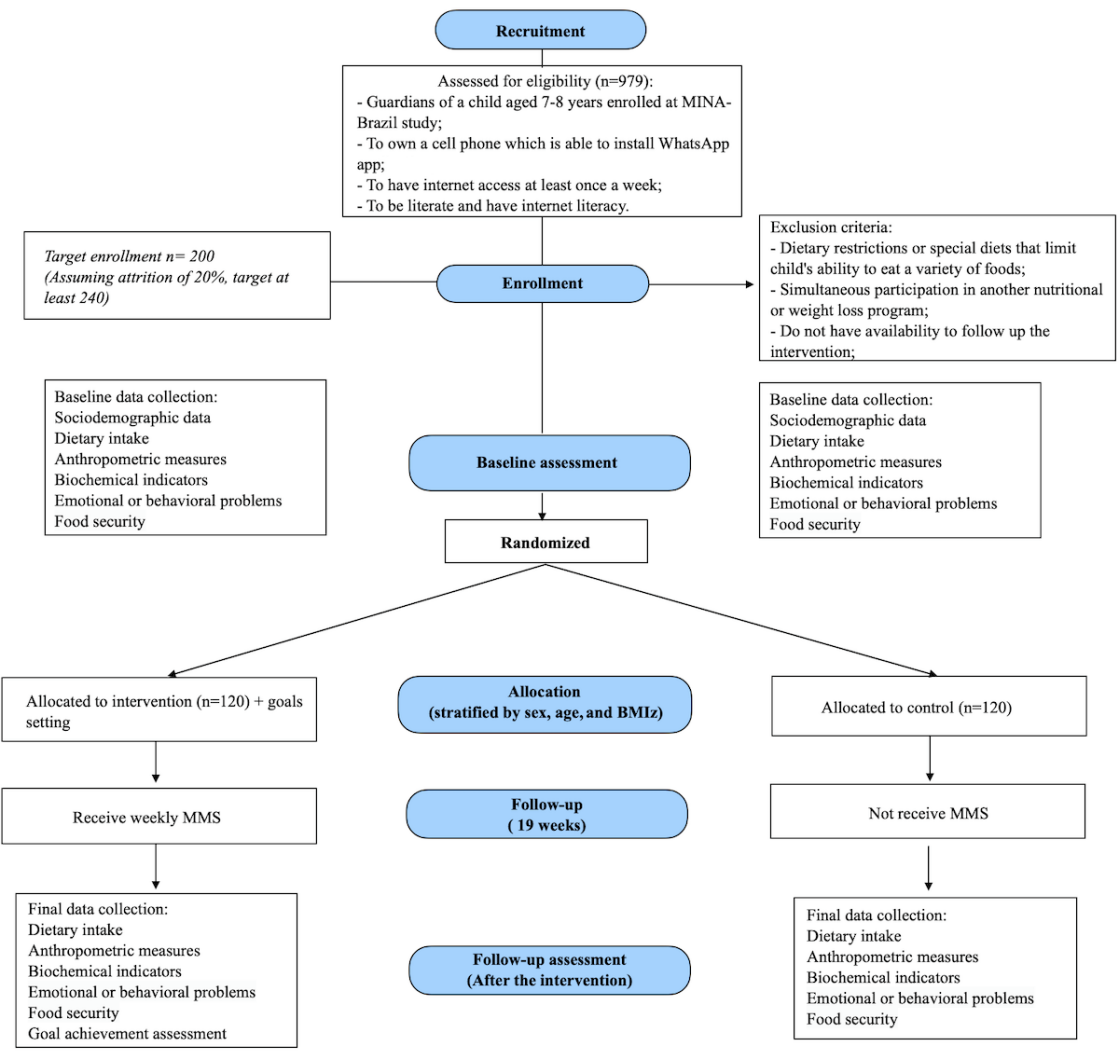
Study flowchart. BMIz: BMI in *z* score; MINA-Brazil: Maternal and Child Health and Nutrition Cohort Study in Acre; MMS: Multimedia Message Service.

We will recruit 300 parents or caregivers of children enrolled in the MINA-Brazil birth cohort (Figure 1). They will be contacted by phone calls made from the Laboratory of Telephone Interviews of the Faculty of Public Health of the University of Sao Paulo. Family phone numbers will be assessed from the MINA-Brazil study database. Trained interviewers will make the calls at different times of the day to maximize the chance of contact with parents or caregivers. Additionally, MINA-Brazil’s social media (ie, Facebook and Instagram) will be used to contact families who have changed their phone numbers but have not updated this information in their records. At this first contact, eligibility criteria will be assessed; then, the study’s aim will be explained, and the parent or caregiver will be invited to be part of the study. Following this, a face-to-face meeting will be scheduled at a health care center in Cruzeiro do Sul with children and parents or caregivers to collect the consent forms and conduct the initial interview.

Inclusion criteria for the study include (1) being a guardian of a child aged between 7 and 8 years enrolled in MINA-Brazil, (2) owning a cell phone that can install the WhatsApp app, (3) having internet access at least once a week, and (4) being literate and having internet literacy.

Exclusion criteria include (1) dietary restrictions or special diets that limit a child’s ability to eat a variety of foods (eg, children on treatment for any disease that requires a specific diet), (2) simultaneous participation in another nutritional or weight loss program, and (3) not having the availability to follow up on the intervention for the whole period between baseline and final data collection (eg, intending to move out of town).

### Power and Sample Size Calculation

The sample size estimation used the expected effect size (E) of at least 10% change for the mean value of BMIz as the main outcome (*z* score=1.5) and SD of 3.5 in a 2-tailed distribution, considering a *P* value of .05, statistical power of 80% (β=20%), and sample size ratio (group 1/group 2) of 1:1. Thus, for the expected E/SD ratio, the minimum sample required must be 100 participants per group [49]. However, assuming a dropout rate and potential losses of 20% during the intervention period, the sample size to be enrolled at baseline will be at least 120 children per study group [49].

### Ethical Considerations

The MINA-Brazil birth cohort was approved by the Ethics Committee in Research with Human Beings of the School of Public Health of the University of São Paulo (protocols 872,613 of November 13, 2014, and 872,613 of October 30, 2017). For this trial, a new protocol was submitted and approved by the same Ethics Committee in Research with Human Beings (protocol 5,805,325 of December 9, 2022). Before taking part in the study, consent forms will be collected from parents or caregivers, and assent forms will be collected from children to guarantee the voluntary nature of participation. Additionally, participants will be informed of the minimal anticipated harms of the intervention, such as the possibility of discomfort related to interviews with personal questions and venous blood collection, which will involve trained professionals and disposable materials. All participants will receive appropriate information on what to do if they wish to withdraw from the intervention at any time.

### Baseline Information and Randomization

Face-to-face individual data collection meetings will take place before the beginning of the intervention to explain the study to parents or caregivers and schedule fasting blood collection at a local laboratory. After recruitment and data collection, participants will be randomly assigned to each experimental group using the R software (R Foundation for Statistical Computing), according to children’s sex, age, and BMIz quartile intervals. Additionally, the stratified sortition will consider parents’ or caregivers’ schooling in years (≤9, 10-12, or ≥13) and wealth index quartiles.

### Blinding

Given the nature of the intervention, we cannot guarantee participants will be blinded. However, study participants will not be aware of which group they will be assigned to. All participants will be informed that they will take part in a nutritional program; however, some children will receive the intervention before others. Furthermore, this strategy aims to minimize the chances of contamination bias due to information exchange between participants in the control and intervention groups. Researchers will be aware of participants’ allocation, but all analyses will be blind to allocation.

### Theoretical Basis

**Figure 2:**
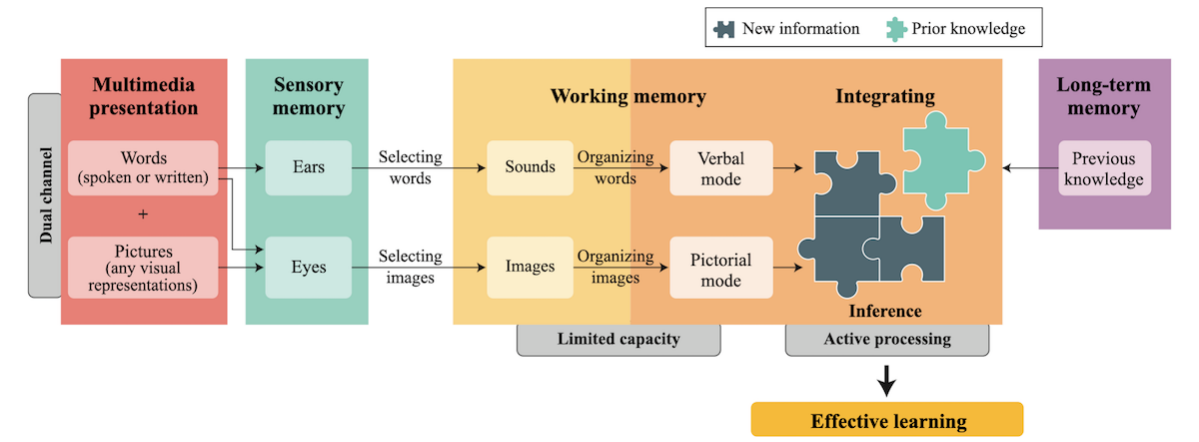
Multimedia information processing system according to the cognitive theory of multimedia learning.

This intervention is grounded in the cognitive theory of multimedia learning (CTML) [50]. The CTML guides the development of efficient multimedia materials and how to implement effective cognitive strategies to amplify learning and promote deep understanding [50]. According to Mayer [50], the use of a dual learning channel activates the verbal and pictorial models of working memory. This activation allows readers to mentally connect visual and textual representations [50,51]. However, to promote effective learning, that is, to reach the individual’s long-term memory, the information provided to the reader must be integrated into the working memory in a process called active processing (Figure 2 [50]). To trigger active processing, it is necessary for readers to form a mental representation connecting new knowledge to preexisting knowledge. In this way, readers will be able to make inferences regarding the combination of these contents to establish the understanding itself. Inferences are a central part of the understanding process, and the more inferences individuals make, the greater their active processing [52]. Furthermore, it is important to consider that working memory has a limited capacity. Thus, readers should not be overwhelmed with a large number of new concepts all at once. Therefore, for effective processing of information, the multimedia content should have as few words as possible and reinforce previously discussed concepts [50,52].

**Figure 3:**
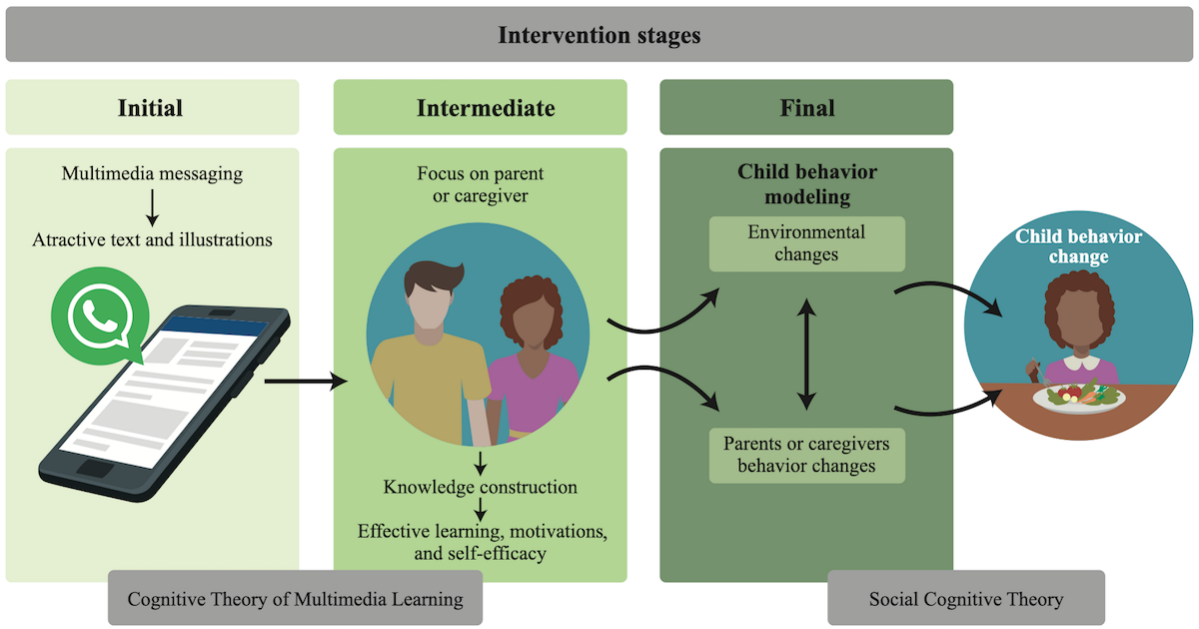
The framework of the intervention.

Also, social cognitive theory (SCT) was adopted as one of the foundations in the conception of the intervention. This theory claims that the behaviors of individuals are influenced by personal characteristics and the environment around them [53]. Thus, Bandura [53] argues that behavioral changes in health can be more effective if they strengthen individuals’ knowledge on the subject, improve environmental factors such as social support, encourage self-efficacy, and, lastly, develop the use of self-regulatory behaviors through modeling of behaviors. Figure 3 shows the framework of the intervention, presenting how multimedia messages will operate to change children’s behavior based on both CTML and SCT.

### Intervention Plan

Participants allocated to the intervention group will receive individual multimedia messages 3 times a week for 19 weeks [54]. The message-sending system will be programmed using the Python programming language [55]. For individual goal-setting, parent-child or caregiver-child dyad will choose 2 specific, measurable, attainable, realistic, and timely (SMART) goals to focus on during the intervention according to their specific needs [56]. The goals will be chosen according to a preestablished list that will be defined by the topics of the messages that will be sent to parents or caregivers [56,57]. In addition, participants will receive an educational booklet about recommended dietary practices based on the food guide for the Brazilian population [58]. One week after the intervention commences, parents or caregivers will be contacted by phone calls to verify if they are appropriately receiving the messages.

### Multimedia Message Content

To elaborate on the textual component of multimedia messages, the following 2 materials will be used as a guide: (1) the food guide for the Brazilian population [58] and (2) the protocol for the use of the food guide for the Brazilian population in the food guidance of children aged between 2 and 10 years [59]. Additionally, to assess the study population’s dietary practices, previous results from a 24-hour Dietary Recall and Food Frequency Questionnaire applied to the target population were screened. While structuring the messages, behavior change techniques and persuasive writing strategies were used. Messages were structured according to 5 main topics related to the intervention objective: (1) increase fresh foods consumption, (2) reduce UPF consumption, (3) reduce culinary ingredients consumption (eg, excessive use of salt, sugar, and deep-frying practice), (4) encourage culinary practices, and (5) promote commensality and a healthy relationship with food. For the design of illustrations, we will adopt simple, precise, and easily recognizable images. In addition, persuasive techniques of color combination, typography, and cultural components, among others, will be applied [50,52].

### Cross-Cultural Adaptation

To ensure the cultural appropriateness of the messages for the target population, the text component will undergo a 2-step cross-cultural adaptation [60]. The first step will include an expert committee of health professionals with experience in care or research in the Amazon region. In the second step, children’s guardians will be invited to participate in formative research to assess the understanding and relevance of the elaborated messages. The formative research will be conducted through a telephone interview using a semistructured questionnaire with 4 exploratory questions for each SMS text message [61]. Then, the answers will be analyzed by 2 researchers, and the necessary adjustments will be made. Parents or caregivers involved in this phase will not be included in the intervention study.

### Control Group

The control group will consist of children from the same population as the intervention group to ensure that both groups share similar characteristics, thereby guaranteeing accurate results for the intervention. In this context, the control group will not receive any messages during the intervention period. However, participants allocated to this group will be informed that they are on a waiting list and that the intervention will be carried out in stages, where children will receive the intervention at different times. After the end of the intervention period, the control group will receive the same treatment as the intervention group [35].

### Outcome Measures

Participants in both groups will have the following data collected at baseline and immediately post intervention. The data obtained will be compiled onto tablets or computers with the Census and Survey Processing System (CSPro) program (US Census Bureau and ICF Macro).

### Primary Outcome (BMIz)

Children in both experimental groups will have their weight and height measured by trained study researchers according to the parameters established by the WHO [62]. BMIz and children’s anthropometric indices, weight for age and height for age, will be assessed through WHO Anthro-Plus software and will be interpreted as recommended by the WHO [63]. In addition, children’s waist circumference (WC) will be measured according to WHO recommendations [64]. Since no WC cutoff points were found in the literature for Brazilian children, to evaluate WC, we will consider that children with WC values above the 90th percentile of the sample have excessive abdominal fat [65,66]. The waist-to-height ratio will also be calculated, in which values above 0.5 will be considered to correspond to the presence of excessive abdominal fat [65,67].

### Secondary Outcomes

#### Dietary Habits

In natural and minimally processed food (fruits, vegetables, grains, tubercles, and cereals) and UPF (chocolate powder, artificial and soft drinks, industrial bread, sweet-filled biscuits, salty crackers, deli products, pre-prepared frozen foods, instant noodles, sweets, and candies, among others), consumption will be assessed through a 1-month validated food frequency questionnaire for Amazonian schoolchildren [68]. Data will be collected by a trained nutritionist, and the frequency of food consumption will comprise the following 8 categories: rarely, 1-3 times a month, 1-3 times a week, 1 time a day, 2-3 times a day, 4-6 times a day, and more than 6 times a day.

#### Biochemical Indicators

Fasting blood collection will be performed by venipuncture to determine the lipid profile (total cholesterol, high-density lipoprotein [HDL], low-density lipoprotein [LDL], and very–low-density lipoprotein cholesterol), insulinemia, and blood glucose in a local clinical analysis laboratory with automated procedures. The lipid profile of the children will be interpreted according to the Brazilian Society of Cardiology, in which values <150 mg/dL for LDL and >45 mg/dL for HDL will be considered acceptable [69]. Blood glucose will be interpreted according to the Brazilian Society of Pediatrics, which establishes adequate values when <100 mg/dL [70]. Additionally, blood glucose and insulinemia will be used to assess peripheral insulin resistance through the Homeostasis Assessment Model-Insulin Resistance Index [71]. Results above the 90th percentile of the sample will indicate the presence of insulin resistance [71].

### Other Measures

#### Sociodemographic and Participant Information

At baseline, only children’s sex, age, and who is their primary caregiver will be assessed. For caregivers, the level of education and wealth index will be measured.

#### Emotional or Behavioral Problems

To screen ’children’s mental health problems, the Strengths and Difficulties Questionnaire (SDQ) will be completed by the children’s parents or caregivers [72]. SQD has 25 items divided between 5 scales: emotional symptoms, conduct problems, hyperactivity/inattention, peer relationship problems, and prosocial behavior [72]. Total difficulty score will be calculated from the summary of the scale results (except prosocial behavior), and the final score will be classified into 3 categories: normal (0-13), borderline (14-16), and abnormal (17-40). For the assessment of prosocial behavior, a higher score will indicate better performance on this scale.

#### Food Security

To assess the food security of the study population, the short version of the Brazilian Food Insecurity Scale [73], with 5 questions, will be used. Study participants will be classified as food secure (0 points) or food insecure (>0 points) [73].

### Data Analysis

The analysis will use an intent-to-treat approach to test the efficacy of the intervention. Tablets programmed with CSPro (US Census Bureau, ICF International) will be used for data entry. Descriptive statistics will be calculated for parents or caregivers and children. Paired and unpaired 2-tailed *t* tests will be applied when comparing mean changes of the outcome variables from pre- to post intervention when examining within-group changes in exploratory data analysis. For skewed distribution data, the Mann-Whitney test will be used to assess differences between groups. Multiple imputation methods will be used to deal with missing data. All analyses will be performed with the statistical package Stata 18.0 or higher (StataCorp), at a significant level of *P*<.05.

## Results

Data collection started in June 2023, and 266 children were enrolled at baseline. Final measurements are scheduled to be completed in December 2023. The results of the main analysis will be conducted in 2024 and are expected to be available in 2024 and 2025.

## Discussion

### Overview

This is the first mHealth intervention focused on Amazonian children and also the first one, as far as we know, that investigates the efficacy of multimedia messages on children’s dietary habits and excessive weight gain. Furthermore, it is novel because it translates policy-level recommendations from the food guide for the Brazilian population to a hard-to-reach and vulnerable population.

Furthermore, due to both food insecurity increases and large consumption of UPF, nutritionally adequate and environmentally sustainable food patterns from local Amazonian communities have been compromised, which may greatly influence global environmental changes [74,75]. Diets based on large consumption of UPF cause higher greenhouse gas emissions through the need for large deforestation areas for monoculture farms and industrial processes. This contributes to biodiversity loss, land degradation, and intensification of climate change [74].

### Conclusion

This trial is expected to better understand the efficacy, challenges, and limitations of the use of technologies in healthy eating promotion, particularly in regions characterized by high social vulnerability and limited access to health care, as is the case in the Western Amazon. We hypothesize that children whose parents or caregivers receive multimedia messages related to nutrition will demonstrate improvements in BMIz, dietary food intake, and biochemical data. Furthermore, we expect that the data produced by this trial will contribute to the development and strengthening of innovative public health policies aimed at preventing childhood obesity.

## Appendix

Multimedia Appendix 1 CONSORT-EHEALTH checklist (V 1.6.1).

## Abbreviations

BMIz: BMI in z score
CSPro: Census and Survey Processing System
CTML: cognitive theory of multimedia learning
E: effect size
HDL: high-density lipoprotein
LDL: low-density lipoprotein
mHealth: mobile health
MINA-Brazil: Maternal and Child Health and Nutrition Cohort Study in Acre
SCT: social cognitive theory
SDQ: Strengths and Difficulties Questionnaire
SMART: specific, measurable, attainable, realistic, and timely
UPF: ultraprocessed food
WC: waist circumference
WHO: World Health Organization

## Data Availability

Data sharing is not applicable to this article as no data sets were generated or analyzed during this study.
